# 

**Published:** 1889-06

**Authors:** 


### American Medical Association.

This body meets June twenty-fifth prox. in Newport, R. I. This will undoubtedly be a large and interesting meeting. The profession of the east will be present in large numbers, as it is at a central point in the east; and many men of the west are in the habit of visiting the East once a year, and many others will desire to visit it to be present at this meeting, and perhaps western men are accustomed more than those of the east to traveling long distances; and besides the President is a Western man and well known to the profession through the country, and it will be very gratifying to him to welcome a large number of its members from his own section of the country.

The various specialties of medicine and surgery have their departments or sections in this meeting, and it is always prophetic of good when each and every one of these is well sustained.

The indications are that the Dental Section will be well attended and that a number of papers on interesting subjects will be read and considered.

The following is the programme, in part at least, of the work of this section:

PROGRAMME.

TUESDAY, JUNE 25TH, 1889.

Address........ ..............................F. H. REHWINKEL.

Facial Neuralgia Associated With Pregnancy.
DR. W. W. ALLPORT.

Pus.............................................DR. WM. H. ATKINSON.

WEDNESDAY, JUNE 26TH, 1889.

Diseases of the Antrum....................DR. WM. CARR.

Paper (title not given)....................DR. J. TAFT.

Fissures ........................................ DR. R. R. ANDREWS.

THURSDAY, JUNE 27TH.

Care of the Teeth of Pregnant Women.DR. JOHN MARSHALL.

Statistics of Irregularity of the Teeth of Normal Individuals, the Idiotic, Deaf, Dumb, Blind, and Insane.
..............................................DR. EUGENE S. TALBOT.

### THE DENTAL REGISTER,

A MONTHLY MAGAZINE DEVOTED TO THE INTERESTS OF THE

**DENTAL PROFESSION.**

*Terms Reduced to* $2.00 *Per Year. Single Copies,* 25 *Cents.*

**EDITED BY PROF. J. TAFT, D.D.S.**

—AND—

**Published by SAM'L A. CROCKER & CO., Ohio Dental and Surgical Depot.**

The volume commences in January and closes in December.

The REGISTER being issued monthly is always fresh, therefore Indispensable to the practitioner who desires to keep posted up with the new inventions and improvements which are daily developed. Neither expense nor effort will be spared to give value and variety to its contents. Articles will be illustrated with well executed engravings whenever they will add to the interest or assist to explanation.

Its extensive circulation and frequency of issue make it one of the BEST DENTAL ADVERTISING MEDIUMS in the United States.

All subscriptions must begin with January or July.

Local or special notices, five lines or less, will be inserted for $3 each insertion; over five lines, 50 cts. per line.

All advertising bills payable in advance, or at furthest, after the issue of the first number containing the advertisement. All transient advertisements to be paid for in advance.

☞CONTRIBUTIONS SOLICITED.

Exclusively Editorial Communications should be addressed to the Editor.

The latest number of the REGISTER may be ordered with or without other goods at any time, and all letters should be addressed to

SAM'L A. CROCKER & CO., Ohio Dental and Surgical Depot, Cincinnati, O.

